# Electromagnetic Vortex-Based Radar Imaging Using a Single Receiving Antenna: Theory and Experimental Results

**DOI:** 10.3390/s17030630

**Published:** 2017-03-19

**Authors:** Tiezhu Yuan, Hongqiang Wang, Yongqiang Cheng, Yuliang Qin

**Affiliations:** School of Electronic Science and Engineering, National University of Defense Technology, Changsha 410073, China; oliverwhq@tom.com (H.W.); nudtyqcheng@gmail.com (Y.C.); qinyuliang@nudt.edu.cn (Y.Q.)

**Keywords:** radar imaging, orbital angular momentum (OAM), vortical radio wave, uniform circular array (UCA), phase compensation, azimuth resolution

## Abstract

Radar imaging based on electromagnetic vortex can achieve azimuth resolution without relative motion. The present paper investigates this imaging technique with the use of a single receiving antenna through theoretical analysis and experimental results. Compared with the use of multiple receiving antennas, the echoes from a single receiver cannot be used directly for image reconstruction using Fourier method. The reason is revealed by using the point spread function. An additional phase is compensated for each mode before imaging process based on the array parameters and the elevation of the targets. A proof-of-concept imaging system based on a circular phased array is created, and imaging experiments of corner-reflector targets are performed in an anechoic chamber. The azimuthal image is reconstructed by the use of Fourier transform and spectral estimation methods. The azimuth resolution of the two methods is analyzed and compared through experimental data. The experimental results verify the principle of azimuth resolution and the proposed phase compensation method.

## 1. Introduction

The orbital angular momentum (OAM) of electromagnetic (EM) waves has been attracting increasing attention in optics [1] and microwave domains [2,3,4] because it provides a new rotational degree of freedom that is distinct from polarization for information transformation [5]. Hitherto, applications of OAM are mainly concentrated in communications [6,7,8,9] in both optical and radio frequencies, which makes use of the orthogonality of different OAM modes. Besides, the peculiar wavefront of EM vortices may be useful for sensing target scenes. Recently, the authors presented the application of the azimuth resolution of EM vortices in radar imaging applications, called EM vortex imaging [10,11,12,13,14].

Most conventional radar imaging techniques obtain two-dimensional target images via Range-Doppler (RD) principle. High range resolution is obtained by using large bandwidth waveform, whereas the high resolution in cross-range needs large aspect-angle variation. High azimuth resolution is usually obtained by increasing the aperture, as in synthetic aperture radar (SAR). The relative motion in SAR achieves angular diversity. The phase front of an EM vortex is not planar, but rotates around the transmission axis [15], showing a helical structure in space. The phase of an EM vortex on a constant range varies with azimuth by an integer multiple *α* of 2π, where *α* is the OAM mode number. As a result, the phase pattern appears as plane waves illuminating from continuous azimuth simultaneously, achieving angular diversity. As radar coincidence imaging uses the stochastic radiation field [16], the helical phase front may provide the OAM-carrying EM waves the ability of azimuth resolution without requiring relative motion [11].

**Figure 1:**
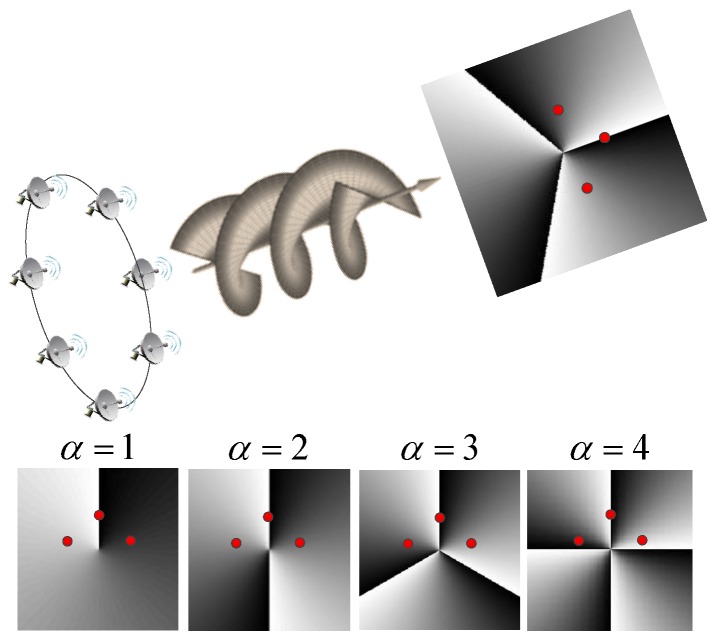
Illustrations of azimuth resolution using vertical radio waves with different orbital angular momentum (OAM) mode numbers. The red points stand for three scatterers with different azimuth angles.

In contrast to the traditional plane wave, the vortical radio wave can provide more information in the cross-range direction. Li et al. [17] applied vortical radio waves to conventional diffraction tomography, achieving imaging resolution beyond the Rayleigh limit. This super-resolution ability derives from illumination of the target with different OAM modes. The principle of azimuth resolution of EM vortex imaging is illustrated in Figure 1, showing the phase patterns of four modes on the cross-section perpendicular to the transmission axis. It is known that the phases on the three scatterers are constant when the plane wave is utilized. The phases on these scatterers are different when vortical radio waves are used. Therefore, the adjacent scatterers with different azimuth angles can be resolved using the echoes of the EM vortex. The azimuthal phase dependency actually achieves angular diversity, as that in SAR is achieved through relative motion. The echo of one OAM mode cannot provide enough information for azimuth resolution. Thus, multiple modes are needed to achieve high azimuthal resolution. The more OAM modes the EM fields carry, the better the azimuth resolution that the images can achieve. A uniform circular array (UCA) can produce vortical radio waves with different modes using appropriate phase shift [5], which has been used for EM vortex imaging but is not the only choice. Range resolution can be achieved by using frequency diversity. When the vortical radio waves with various modes and frequencies are used, the range-azimuth images can be reconstructed from the two-dimensional echoes.

Previous studies mainly used array antennas as receivers, where all the elements receive the echo with the same phase shift as transmitting [11]. In fact, the echo received by a single antenna can also achieve azimuth resolution. In this paper, we focus on EM vortex imaging with a single antenna as the receiver. We first formulate the imaging model with a single receiver located at an arbitrary position. Compared with the multiple-receivers case, the target images cannot be reconstructed directly by Fourier transform method due to the sign changes in the envelope of the received signal, which leads to a point spread function (PSF) without a peak. We propose a method to remove the sign changes in the echo envelope; that is, compensating a phase for each OAM mode based on the array parameters and the elevation of the target. A proof-of-concept imaging system is created and experiments are performed in an anechoic chamber. The phase compensation method is detailed, and further analysis of the experimental results is done in this paper. The simulation and experimental results validate the imaging principle and the effectiveness of the proposed phase compensation method.

The rest of this paper is organized as follows. In Section 2, the imaging model and PSF are compared for the single and multiple receiving antennas, and the failure of the basic Fourier method is revealed. Section 3 details the proposed phase compensation method. Section 4 introduces the experimental setup and gives the azimuthal image of the corner-reflector target reconstructed by different methods using the experimental data. Finally, we conclude this paper.

## 2. Imaging Model and Problem Statement

The considered EM vortex imaging system consists of a vortex beam transmitter and a single receiver. Since it needs multiple OAM modes for azimuthal resolution, a circular phased array antenna is used as the transmitter. It is convenient to generate various modes by feeding with different phase shifts.

**Figure 2:**
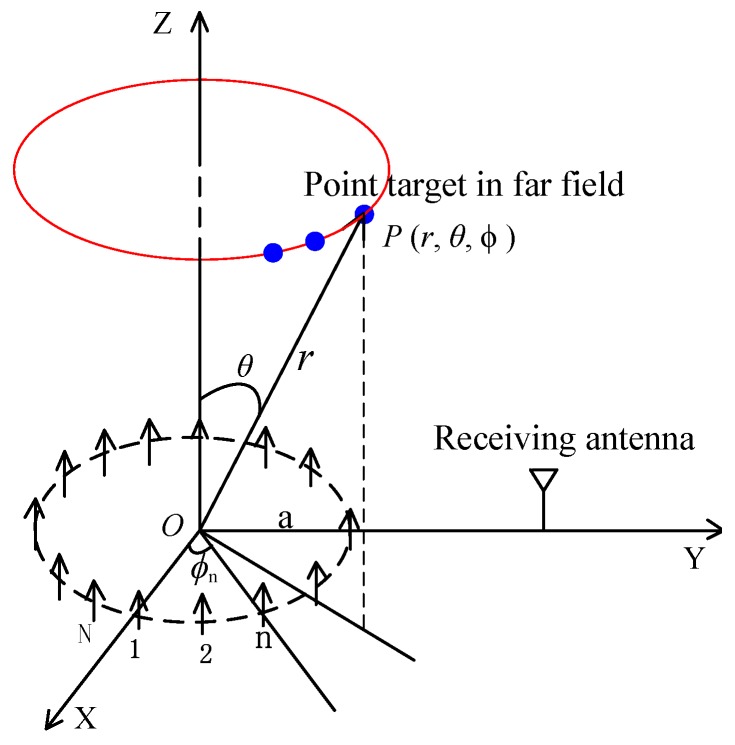
Coordinate frame of electromagnetic (EM) vortex imaging.

The typical imaging geometry is presented in Figure 2. According to the phase pattern of the vortical radio waves [15], the phase varies linearly along the azimuthal direction (the red circle in Figure 2) within the beam. When the target is illuminated by the vortical EM waves, its azimuth information is modulated on the OAM dimension, which can be extracted from the echoes of multiple OAM modes. The imaging model is formulated in the following.

The transmitting UCA in Figure 2 consists of *N* omnidirectional antennas placed on the circle with radius *a*. Each element is fed a monochromatic signal with wavelength *λ*. To generate mode *α*, the phase shift corresponding to the *n*th element is $\varphi_{n}=\alpha \phi_{n}=2\pi \alpha n/N$. The maximum OAM mode generated by the array follows the Nyquist criterion (i.e., $\alpha_{m}<N/2$, where $\alpha_{m}$ is the maximum mode). The receiver can be placed in any position, which will affect the range profile. For the convenience of implementation and to reduce the effect on range profile, it should be located near the transmitter. For an arbitrary point P(r,θ,ϕ) in the far field, the normalized electric field can be given by [11]:

(1) $$\begin{array}{cc} E(r) & =\sum_{n=0}^{N-1}\frac{1}{|r-r_{n}|}e^{jk|r-r_{n}|}e^{j\varphi_{n}}\\ & \approx \frac{e^{jkr}}{r}\sum_{n=0}^{N-1}e^{-j(k\hat{r}\cdot r_{n}-\alpha \phi_{n})}\\ & \approx Nj^{-\alpha }\frac{e^{jkr}}{r}e^{j\alpha \phi }J_{\alpha }\left(ka\sin\theta \right), \end{array}$$

where k=2π/λ and $J_{\alpha }$ is the Bessel function of the first kind of order *α*. r and $r_{n}$ are the position vectors of point P and the *n*th element, respectively.

Assume the extended target consists of *M* ideal scatterers with backscattering coefficient $\sigma_{m}$, whose positions are denoted by $\left(r_{m},\theta_{m},\phi_{m}\right)$. When the multiple frequencies and OAM modes are employed, the echoes received by the single antenna read

(2) $$S_{r}^{s}\left(\alpha ,k\right)=Ne^{-j\frac{\alpha \pi }{2}}\sum_{m=1}^{M}\frac{\sigma_{m}}{r_{m}r_{m}^{\prime }}J_{\alpha }\left(ka\sin\theta_{m}\right)e^{jk(r_{m}+r_{m}^{\prime })}e^{j\alpha \phi_{m}},$$

where $r_{m}^{\prime }$ is the distance from the *m*th scatterer to the receiver.

When all the elements are used to receive the echoes, the phase shift $e^{j\alpha \phi_{n}}$ is applied to the *n*th element, and then all the echoes are summed coherently. The target echo reads

(3) $$S_{r}^{m}\left(\alpha ,k\right)=N^{2}e^{-j\alpha \pi }\sum_{m=1}^{M}\frac{\sigma_{m}}{r_{m}r_{m}^{\prime }}J_{\alpha }^{2}\left(ka\sin\theta_{m}\right)e^{jk(r_{m}+r_{m}^{\prime })}e^{j2\alpha \phi_{m}}.$$

Seen from the imaging model of Equation (2), the range and azimuth of the target are associated with the wave number *k* and the OAM mode *α*, respectively. Note that there is no elevation resolution because the elevation of the target appears in the envelope of the echoes, which has no effect on the phase. Clearly, the two-dimensional Fourier transform method may be used to reconstruct the range-angle image. However, the amplitude modulation of the Bessel function must be considered, which will affect the azimuth profile.

The azimuth profiles of the PSF of the Fourier transform operator in the two receiving cases are given by

(4) $$\begin{array}{cc} J_{\alpha }^{s}\left(\phi \right) & ≜\int_{-\alpha_{m}}^{\alpha_{m}}J_{\alpha }\left(ka\sin\theta \right)e^{-j\alpha \phi }d\alpha , \end{array}$$

(5) $$\begin{array}{cc} J_{\alpha }^{m}\left(\phi \right) & ≜\int_{-\alpha_{m}}^{\alpha_{m}}J_{\alpha }^{2}\left(ka\sin\theta \right)e^{-j\alpha \phi }d\alpha , \end{array}$$

where $\alpha_{m}$ is the maximum OAM mode used for imaging.

When kasinθ≫1, the Bessel function can be approximated as

(6) $$J_{\alpha }\left(ka\sin\theta \right)\approx \sqrt{\frac{2}{\pi ka\sin\theta }}\cos\left(ka\sin\theta -\frac{\alpha \pi }{2}-\frac{\pi }{4}\right).$$

Based on the FFT property, the PSF in Equations (4) and (5) will possess a pair of symmetric peaks. The Hilbert transform can be used to eliminate the effect of imaging ambiguity [11]. Subsequently, the range-angle images can be directly reconstructed by Fourier method. However, the condition of kasinθ≫1 means that the target is far from the beam axis, which is inapplicable in practice for two reasons. Firstly, according to the radiation patterns of the vortical radio waves, the target will be far from the main-lobe region [15], where the radiation energy is limited and the transmitting power is wasteful. Secondly, there exists mixed mode in the large-elevation region, which will produce ambiguity in the azimuth profile. The way to reduce the ambiguity may spend many antenna elements.

**Figure 3:**
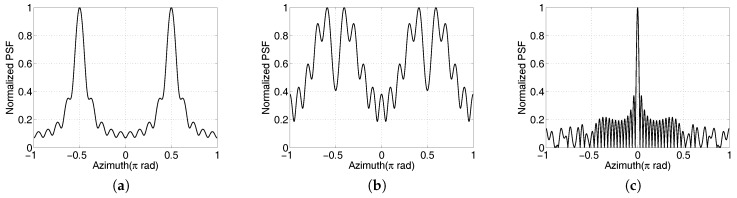
Azimuth profiles of the point spread function (PSF) of the Fourier transform operator with $\alpha_{m}=10$ and ka=10π. (**a**) Large elevation angle (0.4π); (**b**) small elevation angle (0.1π); (**c**) small elevation angle but multiple receivers.

Therefore, a small elevation angle should be chosen in practice, and the approximation of Equation (6) cannot be satisfied. The PSFs of Equation (4) with two different elevation angles are shown in Figure 3. Compared with the large-elevation case, the PSF possesses no peak in the case of small elevation angle. As a result, the Fourier method will fail to construct the target image. However, the PSF in the multiple-receivers case will also possess a peak at zero position, shown in Figure 3c. We focus on the imaging process in the case of small elevation angle when a single receiver is used.

## 3. Phase Compensation and Image Reconstruction

As seen from Equations (2) and (3), the critical difference between the multiple-receiver and the single-receiver cases is that the echo envelope is a Bessel function in the former case and a square Bessel function in the latter. The sign changes in the envelope of the received signal in Equation (2) lead to a PSF without a peak at zero azimuth, whereas the peak in Figure 3c is not affected. If the sign changes of the Bessel function are removed, a similar PSF in Figure 3c can be obtained.

The sign changes mean that the phase is associated with *α* through $J_{\alpha }\left(ka\sin\theta \right)$. When $J_{\alpha }\left(ka\sin\theta \right)<0$ is satisfied, the envelope can be expressed as

(7) $$J_{\alpha }\left(ka\sin\theta \right)=e^{j\pi }\left|J_{\alpha }\left(ka\sin\theta \right)\right|.$$

Clearly, the phase can be compensated by multiplying a phase term

(8) $$\Psi_{\alpha }=\left\{\begin{array}{cc} e^{j\pi }, & J_{\alpha }\left(ka\sin\theta \right)<0\\ 1, & J_{\alpha }\left(ka\sin\theta \right)>0. \end{array}\right.$$

Seen from Equation (8), the phase compensation can be achieved based on the symbol of the Bessel function, which depends on the argument kasinθ and OAM mode *α*. When the array parameters are determined, the phase changes with the elevation angle of the target. Table 1 gives an example of the desired phases for the different modes. A phase shift based on the elevation angle should be added to the echo of different modes. After the phase compensation process, the echo amplitude modulation is similar to the square Bessel function, and the PSF will possess a peak at zero point, which is expressed as

(9) $$J_{\alpha }^{s}\left(\phi \right)=\int_{-\alpha_{m}}^{\alpha_{m}}\left|J_{\alpha }\left(ka\sin\theta \right)\right|e^{-j\alpha \phi }d\alpha .$$

**Figure 4:**
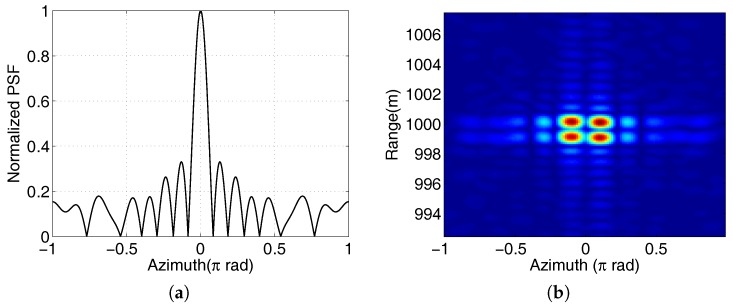
(**a**) The azimuth profile of the PSF of Figure 3b after phase compensation; (**b**) Two-dimensional imaging results of four ideal scatterers whose range and azimuth interval are 1 m and 0.2π, respectively. The signal bandwidth and OAM mode range are 200 MHz and [−7,7], respectively. The array radius is 5λ and the elements number is N=16.

Then, the two-dimensional Fourier transform method can be used to reconstruct the target image. Figure 4 shows the PSF and the simulated imaging result after phase compensation. Clearly, the PSF can provide azimuthal resolution. There are obvious side lobes in both range and azimuth profiles, which can be partly reduced by the windowing technique. Compared with the multiple-receiver case, the azimuthal resolution using a single receiver degenerates, which is

(10) δϕ=2π/Δα,

where Δα is the variation range of the OAM mode. The azimuthal resolution can be improved by using more OAM modes. The range resolution depends on the signal bandwidth.

Seen from Equation (2), the elevation information of the target is contained in the amplitude so that the elevation difference will not affect the PSF. The problem is that the proposed phase compensation method depends on the target elevation. Since the target may extend in the elevation direction, it is difficult to perform phase compensation for all scatterers at the same time. Simulation analysis indicates that this method can tolerate the phase compensation error of two modes, and the main-lobe width of the PSF will not be affected. For the simulation parameters in Figure 4b, the tolerable elevation difference is 5^o^. This imaging scheme cannot obtain the elevation information of the target. It can be obtained by many mature technologies using the same circular array with OAM mode α=0. The position of the receiver has an effect on the range profile if it is far away from the transmitting array. Thus, the receiver should be as close to the array as possible. Ideally, the receiver is located on the center of the transmitting array.

## 4. Experimental Results

In this section, we use the experimental data to verify the proposed phase compensation method and the azimuthal resolution. It is well known that the range resolution is realized by using wideband signal. The azimuthal resolution experiment is performed up to now.

### 4.1. Experimental Configuration

**Figure 5:**
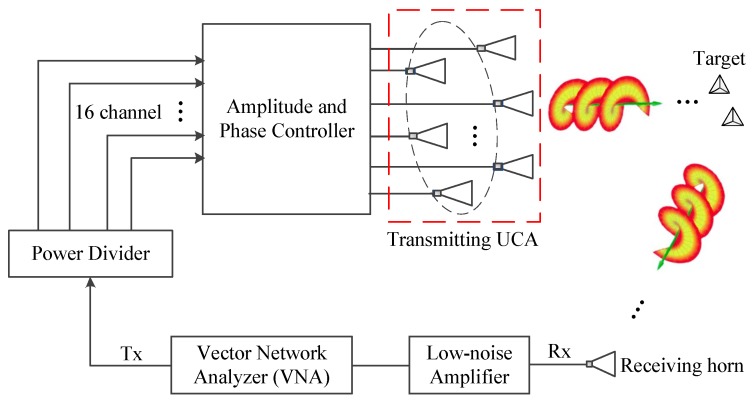
Block diagram of EM vortex imaging system. UCA: uniform circular array.

The imaging system consisted of a vortical radio beam generator and a single receiver. A block diagram related to the experimental system is shown in Figure 5. The vector network analyzer (VNA) was set to transmit and receive the continuous waveform with the monochromatic frequency of 9.9 GHz. The signal produced by the VNA was divided into N=16 parts with equal power. The phase of each channel was adjusted by the phase controller to generate the desired mode. An amplitude controller was used to balance the amplitude of each channel. The antenna array was made up of 16 X-band standard horn antennas with a radius of 5λ. The target echoes were received by a high-gain horn antenna and low-noise amplifier and then measured by the VNA.

The maximum mode generated by this array was seven according to the relationship α<N/2 [5]. The OAM beam with the integer mode between −7 and 7 was generated by feeding with proper phase shifts and received sequentially in time. Then, the echo of each mode was compensated with $e^{-j\alpha \pi /2}$ and the phase associated with the target elevation using the proposed method. The background return was excluded before imaging process. Finally, the azimuthal profile was obtained by applying fast Fourier transform (FFT). Each channel was calibrated to satisfy the phase error below 5 degrees and the amplitude loss below 1 dB.

**Figure 6:**
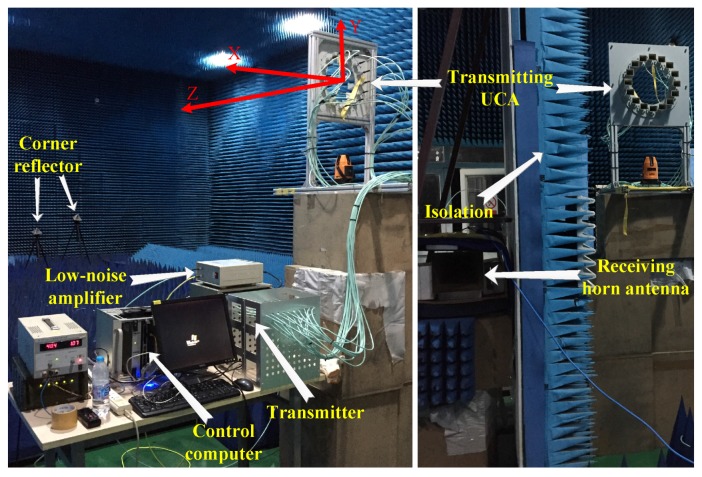
Measurement setup in an anechoic chamber.

The experimental scenario is presented in Figure 6. Measurements were made in an anechoic chamber facility. The center of the transmitting array was 2.3 m above ground level. The receiving antenna was about 1.1 m from the transmitting array. Since a continuous wave was transmitted, the transmitting and receiving antennas were isolated by the microwave absorbing materials to reduce the strong direct echoes. The target—two corner reflectors—was about 8 m from the array. According to the array configuration, the Cartesian coordinates are established in Figure 6 using the red axis.

### 4.2. Results

**Figure 7:**
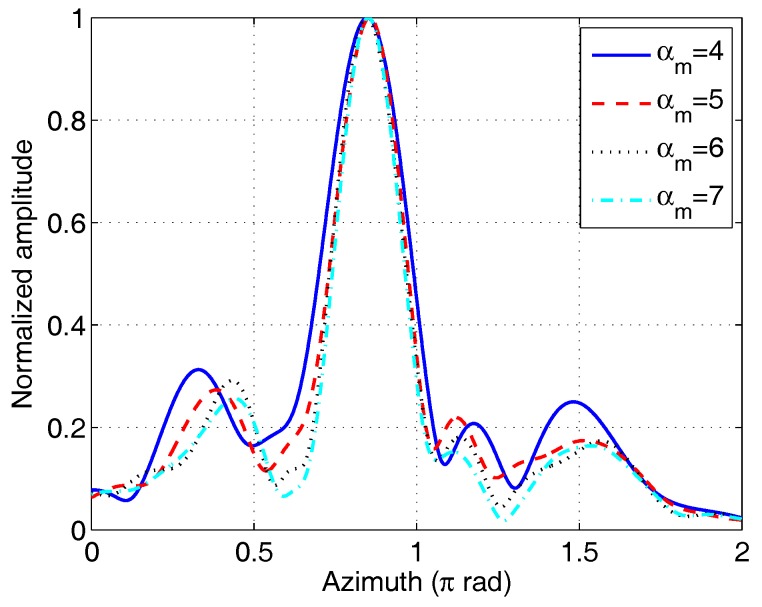
Azimuthal images of 1♯ corner-reflector target using different OAM mode ranges.

The first experiment was performed to verify the relationship between the azimuthal resolution and the OAM mode range. A single corner reflector was placed at (8m,0.05π,0.85π) in a spherical coordinate system, denoted 1♯ corner reflector. Figure 7 shows the imaging results of a single corner reflector. In Figure 7, $\alpha_{m}$ is the maximum OAM mode used for image reconstruction, and each curve corresponds to the OAM modes belong to $\left[-\alpha_{m},\alpha_{m}\right]$. It is evident that the peak appears at the azimuth of the target. The main-lobe width decreased with increasing OAM mode ranges. Because the mode ranges differ slightly, the main-lobe widths of different curves in Figure 7 have small difference. The 3 dB main-lobe width is given in Table 2 along with the theoretical value, 0.886×2π/Δα. The experimental main-lobe width is a little wider than the theoretical width. The sidelobe level of the experimental results conform to the PSF in Figure 4a, which should be further reduced in practical applications.

**Figure 8:**
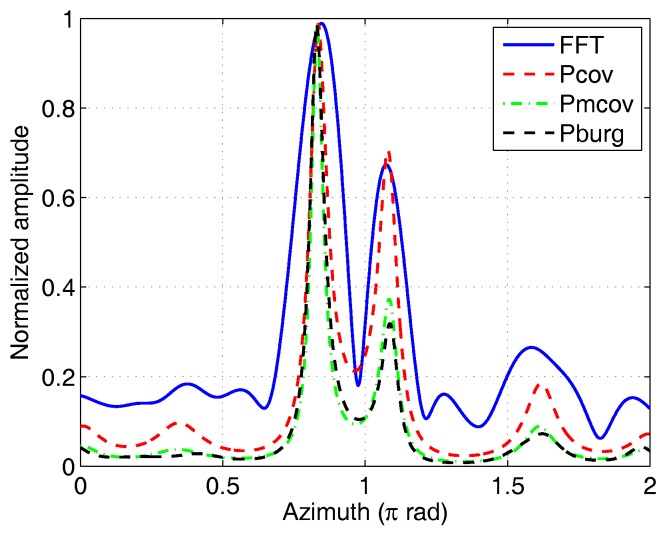
Azimuthal images of two corner reflectors reconstructed by the different methods. FFT: fast Fourier transform method; Pcov: covariance method; Pmcov: Modified covariance method; Pburg: Burg method.

**Figure 9:**
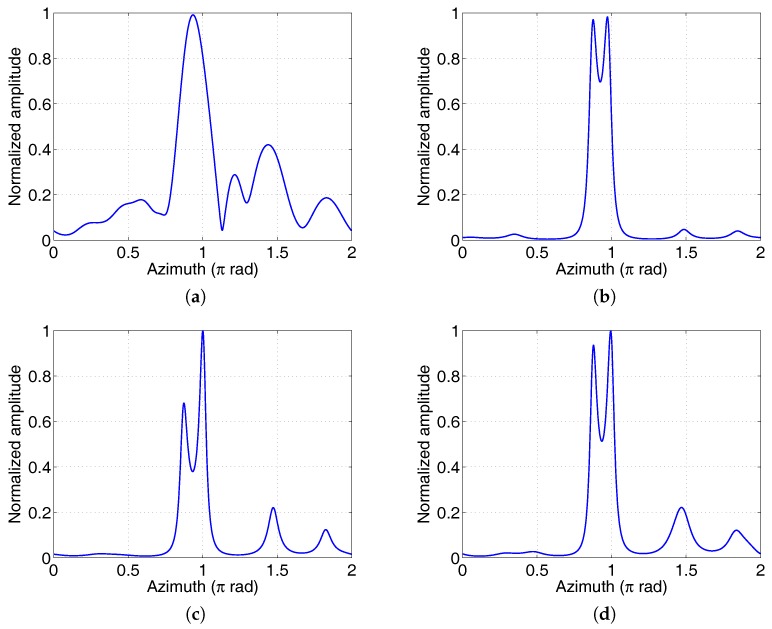
Azimuthal profiles of two corner reflectors using different imaging methods. The distance between the two targets was 0.39 m—half of the Rayleigh limit of the circular array. (**a**) Fourier method; (**b**) Covariance method; (**c**) Modified covariance method; (**d**) Burg method.

In the second experiment we compared the azimuthal resolution of the four imaging methods. The maximum OAM mode $\alpha_{m}=7$ was used for all the imaging methods. Of course, a large OAM mode range will obtain a high azimuthal resolution. The 2♯ corner reflector was located at (8m,0.05π,1.05π), which guarantees that the azimuth interval of the two corner reflectors is 0.2π. The two targets can be resolved by Fourier method based on the results in Table 2. According to the imaging model in Equation (2), other spectral estimation methods can be used for image reconstruction. Figure 8 shows the imaging results of four different spectral estimation methods: Fourier method, covariance method, modified covariance method, and Burg method. As expected, the Fourier method could resolve the two targets. The scattering strength of the 2♯ corner reflector was lower than another. Obviously, the parametric methods could achieve higher resolution than the Fourier method. In contrast, the modified covariance and Burg methods could achieve low sidelobes, but the peak of the weak target was also suppressed. The position of the peak deviated from the real azimuth by about 0.02π rad due to the interaction between the two peaks and the measurement error. In fact, the distance between the two corner reflectors was 0.77 m—a little smaller than the imaging resolution based on the Rayleigh limit (i.e., λr/D = 0.81 m, where *D* is the array aperture). The EM vortex imaging can achieve imaging resolution beyond the Rayleigh limit. This is visible if we decrease the distance of the two targets further. The circular array and the experimental parameters are the same as that in Figure 8, but the azimuths of the two corner reflectors were set to 0.9π and *π*, respectively. The imaging results using different methods are shown in Figure 9. It can be seen that the Fourier method fails because the target distance is less than the resolution decided by the real aperture. Other spectral estimation methods can achieve super resolution and have lower side lobes than the Fourier method. The side lobes of the spectral estimation methods are decided by the model orders.

## 5. Conclusions

This paper investigates a new radar imaging technique using EM waves carrying OAM with a single receiver. This imaging scheme can achieve imaging resolution beyond the Rayleigh limit without motion limitation using spectral estimation methods. The potential of super-resolution in the azimuth direction relies on the fact that the phase front exhibits a helical structure in space, which achieves angular diversity. In contrast to using multiple receivers, the echoes received by a single receiver cannot be used for image reconstruction directly. The phase compensation method is proposed to pre-process the raw data, which is effective when the elevation difference is not too large. The experimental results validate the azimuthal resolution and the proposed phase compensation method. The two-dimensional imaging experiment is ongoing, and an imaging experiment of a metal cube will be performed in the future.

## Figures and Tables

**Table 1:** Phase compensation for different modes and elevation angles with ka=10π.

	*α*	−5	−4	−3	−2	−1	0	1	2	3	4	5
*θ*												
8${}^{\circ }$		*π*	0	*π*	0	0	*π*	*π*	0	0	0	0
9${}^{\circ }$		*π*	0	*π*	0	0	*π*	*π*	0	0	0	0
10${}^{\circ }$		*π*	0	*π*	*π*	0	*π*	*π*	*π*	0	0	0
11${}^{\circ }$		*π*	0	*π*	*π*	0	0	*π*	*π*	0	0	0
12${}^{\circ }$		*π*	0	0	*π*	0	0	*π*	*π*	*π*	0	0

**Table 2:** Comparisons of main-lobe width using different mode ranges.

OAM Mode	Theoretical (Rad)	Experiment (Rad)
[−4,4]	0.22π	0.23π
[−5,5]	0.18π	0.18π
[−6,6]	0.15π	0.17π
[−7,7]	0.13π	0.15π
